# Trends in hypertension- and dementia-related mortality in the United States: An observational analysis from 1999 to 2020

**DOI:** 10.1097/MD.0000000000046611

**Published:** 2025-12-19

**Authors:** Asad Ali Ahmed Cheema, Muhammad Ibrahim, Areen Zia, Umair Iqbal, Ahmed Hasan, Rameesha Babar, Muhammad Moghis, Hurriyah Akbar, Tayyaba Shahiq, Asma Rizwan

**Affiliations:** a International School of Medicine, International University of Kyrgyzstan, Bishkek, Kyrgyzstan; b Department of Medicine, Bahria University Medical and Dental College, Karachi, Sindh, Pakistan; c Department of Medicine, Dow Medical College, Dow University of Health Sciences, Karachi, Sindh, Pakistan; d Department of Medicine, CMH Multan Institute of Medical Sciences, Multan, Punjab, Pakistan; e Department of Medicine, Jinnah Sindh Medical University, Karachi, Sindh, Pakistan; f Department of Medicine, Bakhtawar Amin Medical and Dental College, Multan, Punjab, Pakistan; g Department of Medicine, King Edward Medical University, Lahore, Punjab, Pakistan; h Department of Medicine, Baqai Medical University, Karachi, Sindh, Pakistan; i Karachi University, Karachi, Sindh, Pakistan.

**Keywords:** age-adjusted mortality rate (AAMR), dementia, gender disparities, hypertension, racial disparities, regional disparities

## Abstract

Hypertension (HTN) has long been linked to an increased risk of cognitive decline, and recent years have seen a concerning parallel rise in deaths related to both HTN and dementia in the United States. This study aimed to assess demographic and regional differences in HTN- and dementia-related mortality from 1999 to 2020. We used data from the Centers for Disease Control and Prevention Wide-Ranging Online Data for Epidemiologic Research (CDC WONDER) database to analyze deaths involving both HTN and dementia. Age-adjusted mortality rates (AAMR) and average annual percent change (AAPC) were calculated and stratified by year, sex, race and ethnicity, age group, and geographic region. From 1999 to 2020, a total of 1,266,684 HTN- and dementia-related deaths were recorded. The AAMR increased from 16.7 in 1999 to 128.2 in 2020 (AAPC = 8.05%, *P* < .001), with notable surges in 1999 to 2001 and 2018 to 2020. Women had higher mortality than men (135.6 vs 113.3). Non-Hispanic (NH) Black individuals had the highest rate (115.5), followed by NH White (78.6) and Hispanic individuals (69.2), with Hispanics showing the steepest relative rise. The greatest increase occurred in adults aged 85 years and above, particularly in the Southern (87.1) and rural (85.8) regions. HTN- and dementia-related mortality continues to rise, highlighting widening demographic disparities and the urgent need for equitable preventive health strategies.

**Key Points**

- Hypertension (HTN) and dementia are leading causes of morbidity and mortality in the United States, with a growing public health burden among older adults. HTN affects nearly half of United States adults and is a modifiable risk factor for cognitive decline and dementia, including Alzheimer disease.
- This study examined 1,266,684 United States deaths from 1999 to 2020 where both HTN and dementia were recorded, using CDC WONDER national mortality data. Trends were stratified by age, sex, race/ethnicity, geography, and rural-urban status.
- Age-adjusted mortality rates (AAMRs) from HTN- and dementia-related deaths increased 7.7-fold from 16.7 per 100,000 in 1999 to 128.2 per 100,000 in 2020. The steepest rise occurred between 1999 and 2001 and between 2018 and 2020.
- Disparities were evident across various demographics and geographic regions. Mortality was highest among individuals aged ≥ 85, NH Black populations, women, and residents in the Southern United States and rural regions.
- Despite medical advancements, HTN- and dementia-related deaths have risen significantly, highlighting the need for targeted prevention, improved hypertension control, and equitable access to care.

## 1. Introduction

Hypertension (HTN) is one of the most prominent cardiovascular diseases (CVD) in the United States (US), with half of adults having high blood pressure (BP) or HTN.^[1]^ It accounts for 131 billion dollars in annual expenditure or 3% of the US national healthcare budget.^[2]^ Unlike other CVDs, the trend in hypertensive mortality was upward between 2000 and 2018.^[3]^ Dementia is one of the most common neurological disorders, with Alzheimer being the most common form.^[4]^ By 2060, around 14 million adults are projected to have Alzheimer disease.^[4]^ HTN is a significant modifiable risk factor for cognitive decline and increases risk for dementia, including Alzheimer disease, with cerebral small vessel disease as one of the most important causes.^[5]^ Observational studies showed that an increase in variability of BP in elderly subjects led to the development of dementia.^[6]^ For every 10 mm Hg increase in systolic BP, dementia risk increases by 22% in ages 40 to 59 years and 8% in ages 60 to 69 years.^[7]^ Investigating this longitudinal relationship is crucial for early interventions and reducing the burden of growing cognitive decline.

Over the past 2 decades, the mortality trends for HTN and dementia have been distinct yet interrelated. Despite medical advancement, HTN-related mortality has continued to pose a significant burden, accounting for or contributing to 685,875 deaths in the US.^[1]^ Similarly, dementia-related mortality has risen, especially in older age groups. It is higher in the non-Hispanic (NH) White population, reflecting an aging population as well as increased recognition of neurodegenerative diseases as a cause of death.^[8]^ While the link between HTN and cognitive decline is well established, the parallel rise in deaths attributed to both conditions remains underexplored. Understanding whether this pattern reflects demographic, geographic, or systemic differences rather than a biological association is critical for guiding public health efforts. Therefore, this study hypothesizes that the increasing co-occurrence of HTN- and dementia-related deaths in the US is unevenly distributed across demographic groups and regions. Specifically, we sought to identify which populations and areas experience the highest mortality burden.

Identifying demographic and regional distributions can help us pinpoint populations at the highest risk, enabling early diagnosis and timely intervention. We analyzed data from 1999 to 2020 to maintain a single, finalized series that uses bridged-race population denominators, which underpin age-adjusted rate calculations during this period.^[9]^ Beginning in 2018 to 2023, CDC WONDER provides a separate finalized series that uses single-race denominators; mixing the 2 series would compromise race/ethnicity comparability and long-term trend estimation.^[10]^ In addition, our analyses included urban–rural stratification; confining the study to 1999 to 2020 preserves consistent denominators and avoids documented county-level rate constraints for single-race queries involving Urbanization categories in the newer series. Therefore, we evaluated demographic and regional differences in HTN- and dementia-related mortality in the US from 1999 to 2020 among adults aged 55 years and older.

## 2. Methods

### 2.1. Patient eligibility and screening

This retrospective, population-based observational study analyzed mortality records related to HTN and dementia in the US using the CDC WONDER database. The Multiple Cause-of-Death Public Use Record Death Certificates were studied to identify records in which both HTN and dementia were mentioned as either contributing or underlying cause of death on nationwide death certificates.^[11]^ Deaths were included if both HTN and dementia were listed as either the underlying or a contributing cause of death. Records listing only one condition without the other were excluded. HTN patients were identified with International Classification of Diseases 10th Revision Clinical Modification (ICD-10-CM) codes (I10 to I15), and dementia patients were identified with ICD-10-CM codes F01 (vascular dementia), F03 (unspecified dementia), and G30 (Alzheimer disease) from the multiple cause of death public use record death certificates. Those aged 55 years or older at the time of death were considered older adults. The cutoff of 55 years was selected to capture populations at higher risk of dementia and to minimize inclusion of younger deaths where dementia diagnosis and coding are less reliable. A flowchart summarizing case selection and exclusion is provided in (Fig. S1, Supplemental Digital Content, https://links.lww.com/MD/Q920). This study did not require approval from the Institutional Review Board (IRB) because it was based on data from a de-identified database provided by the government for public use. The STROBE guidelines were followed in this observational study.^[12]^

### 2.2. Data abstractions

Data on deaths due to coexisting HTN and dementia, including population size and location, were gathered between 1999 and 2020. We restricted analyses to 1999 to 2020 to use one finalized dataset with bridged-race denominators (CDC WONDER standard for that period).^[9]^ The finalized 2018 to 2023 series instead uses single-race denominators.^[10]^ Because bridged-race and single-race population estimates differ by construction, pooling them would impair race/ethnicity comparability and bias trend estimates. Moreover, although Urbanization categories are present in both series, the single-race series documents county-level rate and population constraints for analyses involving Urbanization categories, complicating long-run urban–rural comparisons. Accordingly, we analyzed 1999 to 2020 only. Demographic information, including sex and race/ethnicity, as well as regional data, encompassing urban-rural classification and state, were collected from 1999 to 2020. Race/ethnicity categories included NH White, NH Black or African American, Hispanic or Latino, NH American Indian or Alaskan Native, and NH Asian or Pacific Islander. These classifications align with those previously used in analyses from the CDC WONDER database. The National Center for Health Statistics Urban-Rural Classification Scheme was used to classify the population into 2 categories based on the 2013 US census: Metropolitan (large metropolitan area [population ≥ 1 million], medium/small metropolitan area [population 50,000–999,999]), and non-metropolitan (population < 50,000). The United States Census Bureau defines the regions as Northeast, Midwest, South, and West.

### 2.3. Data analysis

The age-adjusted mortality rates (AAMR) were calculated by considering factors such as year, sex, race/ethnicity, state, census region, and metropolitan status. Because the database encompasses all registered US deaths, a sample size calculation was unnecessary. Records with missing or suppressed demographic data (<0.5%) were excluded from subgroup analyses but retained in total counts. This is because the CDC WONDER has population sizes for every demographic, regional factors, and age group for a specific year. To examine the changes over time in mortality rates, we applied the Joinpoint Regression Program (Version 5.0.2, National Cancer Institute).^[13]^ This statistical approach involved fitting log-linear regression models to crude data patterns to calculate the annual percent change (APC) in AAMR, along with its 95% CIs. APCs were classified as increasing or decreasing if the slope explaining the deviation in mortality was markedly different from zero using a 2-tailed t-test with a threshold of *P* < .05.

### 2.4. Sensitivity analysis

As a robustness check to address potential misclassification of underlying vs contributing causes on death certificates, we repeated all primary analyses using a restricted definition in which HTN was required to be the underlying cause of death, and dementia could appear as a multiple (contributing) cause. Estimates were then compared with the main definition (HTN and dementia listed as underlying or contributing causes). Consistency in direction and magnitude across definitions was interpreted as evidence of robustness.

### 2.5. Bias, data source rationale, and confounders

Mortality data from death certificates may be subject to misclassification, because assignment of underlying and contributing causes depends on certifier judgment. We mitigated this by using standardized ICD-10 codes across all years, ensuring that deaths were tabulated based on unique mortality records reporting both conditions, thereby avoiding overlap in categorization, and conducting the sensitivity analysis described above that required HTN as the underlying cause and allowed dementia as a multiple cause. Reliance on death certificates is justified because they are a comprehensive, legally mandated national census of deaths, provide uniform ICD-10 coding, and are the only source of finalized US. mortality counts for the study period. Although CDC WONDER enables stratification by age, sex, race and ethnicity, region, and urban–rural status, it does not contain person-level data on comorbidities, socioeconomic status, or healthcare access; therefore, residual confounding is possible and results should be interpreted as descriptive associations rather than causal effects.

## 3. Results

Between 1999 and 2020, 1,266,684 deaths involving both HTN and dementia were recorded in the US (Fig. S2 and Table S1, Supplemental Digital Content, https://links.lww.com/MD/Q920). Over half of these deaths occurred in nursing facilities (55.4%), followed by homes (18.6%) and hospitals (16.1%) (Table S2, Supplemental Digital Content, https://links.lww.com/MD/Q920).

### 3.1. Annual trends in HTN and dementia-related AAMR

**Figure 1.**
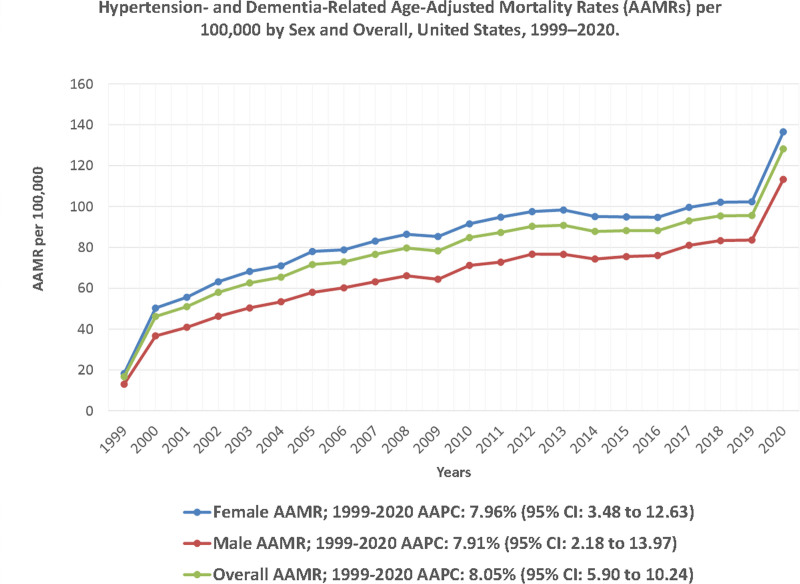
Hypertension- and dementia-related age-adjusted mortality rates (AAMRs) per 100,000 by sex and overall, United States, 1999–2020. Trends in AAMRs are presented for males and females and overall, with joinpoint-derived annual percent change (APC) and 95% confidence intervals shown for each trend segment. AAMR = age-adjusted mortality rate, AAPC = average annual percent change, CI = confidence interval.

The number of deaths due to HTN and dementia increased from 9614 in 1999 to 113,417 in 2020. This represents an absolute increase of more than 103,803 deaths and a relative rise of 1080% across the study period. The AAMR increased 7.7-fold over the 21 years, from 16.7 per 100,000 (95% CI: 16.3 to 17.0) in 1999 to 128.2 per 100,000 (95% CI: 127.5 to 129.0) in 2020, with an average annual percent change (AAPC) of 8.05% (95% CI: 5.90 to 10.24, *P* < .001). Joinpoint regression identified 4 distinct phases in mortality trends. Between 1999 and 2001, AAMR increased sharply from 16.7 to 51.0 per 100,000 (APC: 54.85%; 95% CI: 24.61 to 92.43, *P* = .001), followed by a slower but sustained rise from 51.0 to 87.3 per 100,000 between 2001 and 2011 (APC: 4.40%; 95% CI: 3.49 to 5.32, *P* < .001). Between 2011 and 2018, the trend plateaued, with no statistically significant change (APC: 0.32%; 95% CI: −0.93 to 1.59, *P* = .586). However, from 2018 to 2020, AAMR surged significantly, rising from 95.4 to 128.2 per 100,000 (APC: 16.05%; 95% CI: 8.14 to 24.53, *P* < .001) (Fig. 1; Tables S3 and S4, Supplemental Digital Content, https://links.lww.com/MD/Q920).

### 3.2. HTN and dementia-related AAMR stratified by gender

AAMR increased among both males and females, but female mortality remained higher throughout the study period. Among females, AAMR rose from 18.3 per 100,000 (95% CI: 17.8 to 18.7) in 1999 to 136.5 per 100,000 (95% CI: 135.5 to 137.5) in 2020, with an AAPC of 7.96% (95% CI: 3.48 to 12.63, *P* < .001). The most significant increase was observed between 1999 and 2001, where AAMR surged from 18.3 to 55.6 per 100,000 (APC: 70.15%; 95% CI: 5.87 to 173.45, *P* = .030), followed by a slower but steady increase from 2001 to 2020 (APC: 2.91%; 95% CI: 2.20 to 3.62, *P* < .001). Among males, AAMR rose from 13.1 per 100,000 (95% CI: 12.5 to 13.6) in 1999 to 113.3 per 100,000 (95% CI: 112.1 to 114.4) in 2020, with an AAPC of 7.91% (95% CI: 2.18 to 13.97, *P* = .006). Unlike females, the early increase between 1999 and 2001 was not statistically significant (APC: 59.26%; 95% CI: −13.71 to 193.94, *P* = .128), but from 2001 to 2020, mortality escalated significantly (APC: 3.58%; 95% CI: 2.80 to 4.36, *P* < .001). By 2020, the female-to-male mortality ratio was approximately 1.2:1, indicating a persistent gender gap in HTN- and dementia-related mortality (Fig. 1; Tables S3 and S4, Supplemental Digital Content, https://links.lww.com/MD/Q920).

### 3.3. HTN and dementia-related AAMR stratified by race/ethnicity

**Figure 2.**
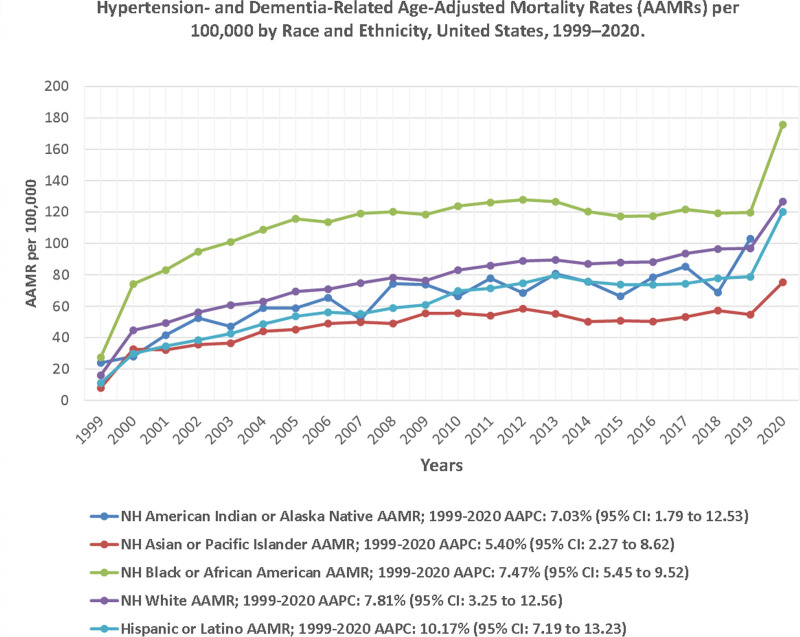
Hypertension- and dementia-related age-adjusted mortality rates (AAMRs) per 100,000 by race and ethnicity, United States, 1999–2020. Trends are shown for non-Hispanic (NH) White, NH Black, Hispanic, NH American Indian or Alaska Native, and NH Asian or Pacific Islander populations. AAMR = age-adjusted mortality rate, AAPC = average annual percent change, CI = confidence interval, NH = non-Hispanic.

Significant disparities were observed across racial groups. NH Black individuals had the highest AAMR, increasing from 27.5 per 100,000 in 1999 to 175.7 per 100,000 in 2020 (AAPC: 7.47%; 95% CI: 5.45 to 9.52, *P* < .001). NH White individuals followed, with a 7.81% annual increase, rising from 16.1 per 100,000 in 1999 to 126.7 per 100,000 in 2020. The Hispanic or Latino population had the steepest relative increase, with AAMR surging from 11.1 per 100,000 in 1999 to 120.1 per 100,000 in 2020 (AAPC: 10.17%; 95% CI: 7.19 to 13.23, *P* < .001). Meanwhile, NH Asian or Pacific Islanders had the lowest AAMR, increasing from 8.0 to 75.3 per 100,000 (AAPC: 5.40%; 95% CI: 2.27 to 8.62, *P* < .001). The absolute racial gap between the highest and lowest groups widened from 19.5 per 100,000 in 1999 to 100.4 per 100,000 in 2020, reflecting growing disparity (Fig. 2; Tables S3 and S6, Supplemental Digital Content, https://links.lww.com/MD/Q920).

### 3.4. HTN and dementia-related CMR stratified by 10-year age group

**Figure 3.**
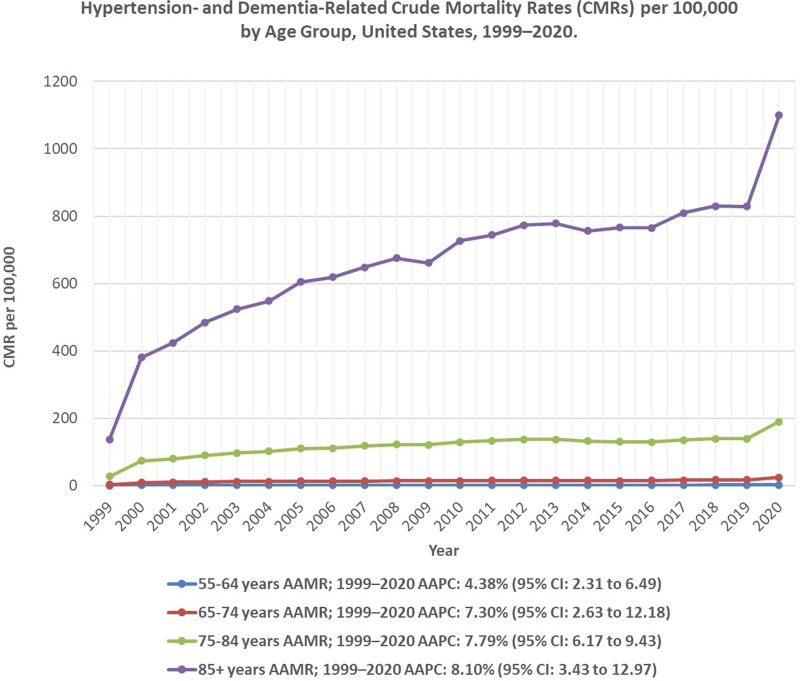
Hypertension- and dementia-related crude mortality rates (CMRs) per 100,000 by age group, United States, 1999–2020. Mortality trends are displayed for age categories 55–64, 65–74, 75–84, and ≥ 85 years. AAMR = age-adjusted mortality rate, CI = confidence interval, CMR = crude mortality rate.

Crude mortality rates increased across all age groups, with the most significant rise occurring in the 85 years and above category. In 55- to 64-year-olds, mortality remained relatively stable between 1999 and 2019, fluctuating between 1.2 and 1.8 per 100,000, before increasing to 2.5 per 100,000 in 2020. In the 65- to 74-year group, mortality increased from 3.1 per 100,000 in 1999 to 17.5 per 100,000 in 2019, followed by a sharp spike to 24.4 per 100,000 in 2020. Among 75- to 84-year-olds, mortality tripled between 1999 and 2003, rising from 27.1 to 97.3 per 100,000, before stabilizing and peaking at 189.9 per 100,000 in 2020. The 85 and above age group experienced the highest CMR, surging from 136.7 per 100,000 in 1999 to 1099.3 per 100,000 in 2020, a more than 700% increase (Fig. 3; Table S5, Supplemental Digital Content, https://links.lww.com/MD/Q920).

### 3.5. HTN and dementia-related AAMR stratified by geographic region

**Figure 4.**
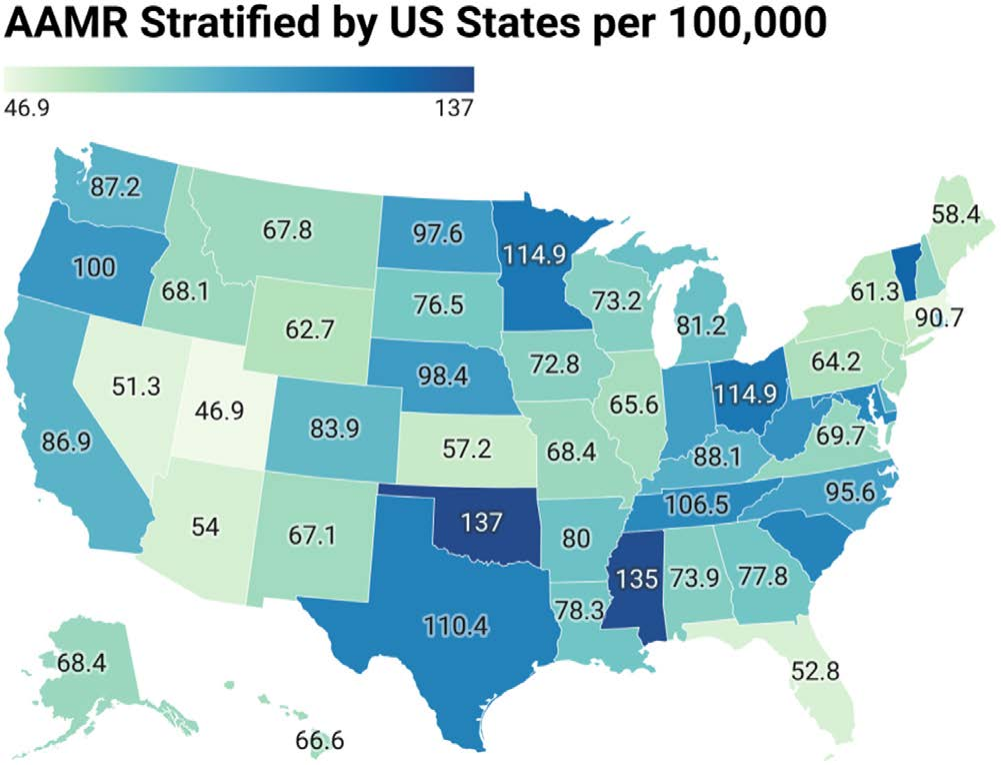
Hypertension- and dementia-related age-adjusted mortality rates (AAMRs) per 100,000 by States, 1999–2020. AAMRs are mapped to illustrate state-level variation in mortality burden. AAMR = age-adjusted mortality rate, CI = confidence interval.

**Figure 5.**
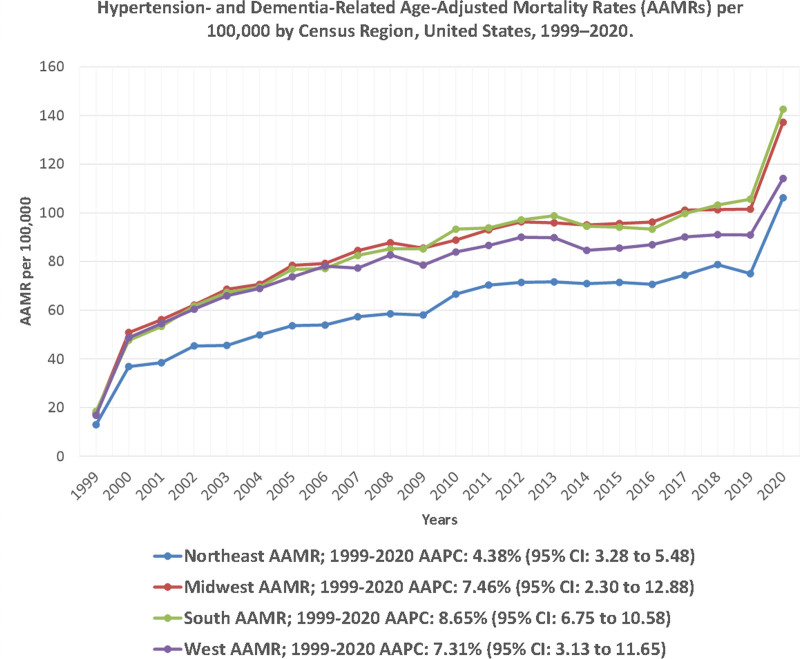
Hypertension- and dementia-related age-adjusted mortality rates (AAMRs) per 100,000 by census region, United States, 1999–2020. Mean AAMRs are shown for the Northeast, Midwest, South, and West census regions, with corresponding 95% confidence intervals. AAMR = age-adjusted mortality rate, CI = confidence interval.

A notable difference in AAMR was observed across all states, with values ranging from 46.9 (95% CI: 45.5 to 48.3) in Utah to 137 (95% CI: 135.3 to 138.6) in Oklahoma. States in the 90th percentile for mortality included Mississippi, Vermont, Ohio, and Texas, while those in the 10th percentile included Massachusetts, Nevada, Florida, and Arizona (Fig. 4; Table S7, Supplemental Digital Content, https://links.lww.com/MD/Q920). Throughout the study period, the highest average mortality was recorded in the Southern region (AAMR: 87.1; 95% CI: 86.8 to 87.3), followed by the Midwestern (AAMR: 85.7; 95% CI: 85.4 to 86.0), Western (AAMR: 80.1; 95% CI: 79.8 to 80.4), and Northeastern (AAMR: 62.4; 95% CI: 62.1 to 62.7) regions. This corresponds to a 39% regional differential between the South and Northeast, persisting across all years (Fig. 5; Tables S7 and S8, Supplemental Digital Content, https://links.lww.com/MD/Q920).

**Figure 6.**
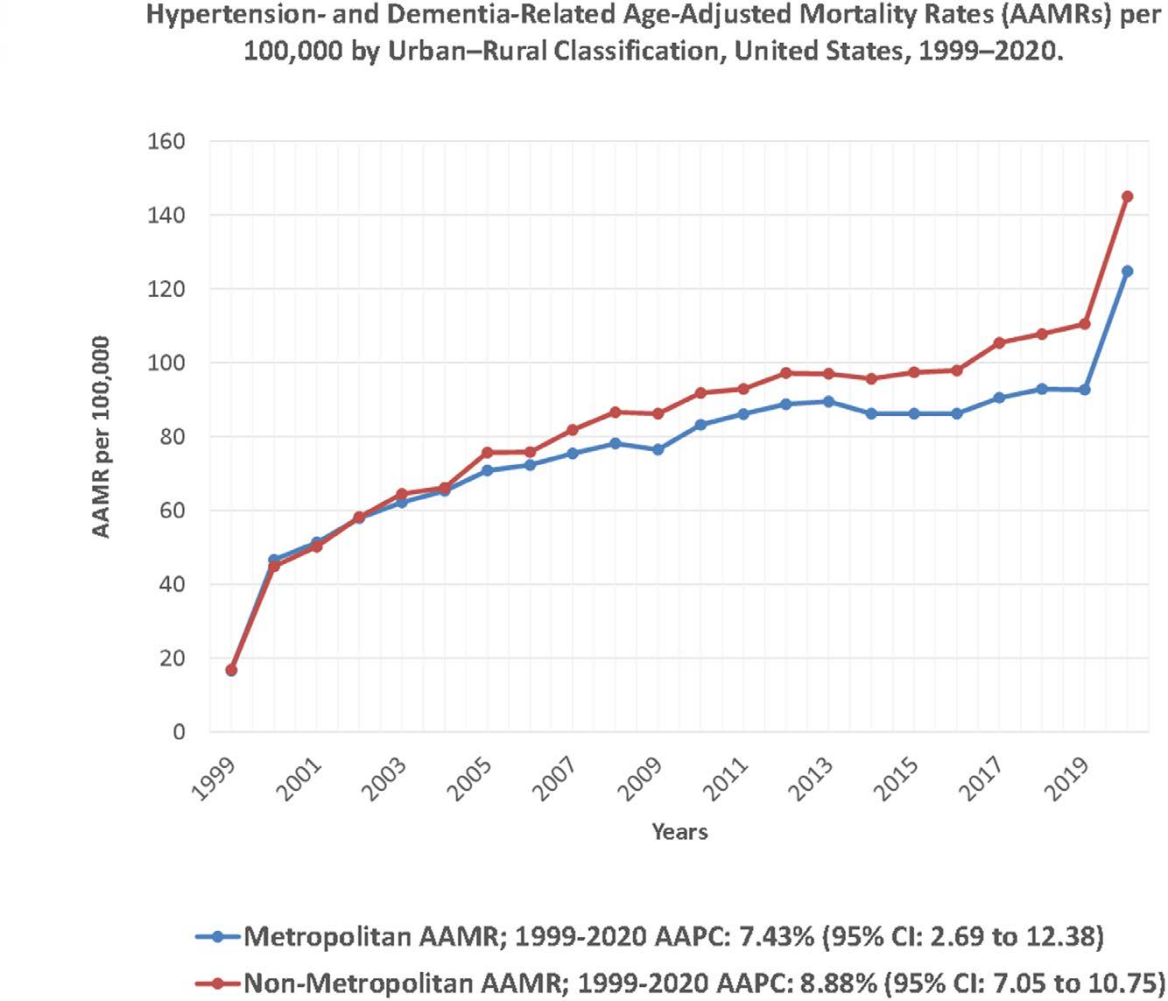
Hypertension- and dementia-related age-adjusted mortality rates (AAMRs) per 100,000 by urban–rural classification, United States, 1999–2020. Trends compare metropolitan (urban) versus non-metropolitan (rural) areas based on the 2013 National Center for Health Statistics classification. All urban–rural comparisons are based on the 2013 NCHS Urban–Rural Classification for Counties and bridged-race denominators (1999–2020) for consistency across the full study period. AAMR = age-adjusted mortality rate, AAPC = average annual percent change, CI = confidence interval, NCHS = National Center for Health Statistics.

Rural areas consistently exhibited higher AAMRs than urban areas throughout the study period, with overall rates of 85.8 (95% CI: 85.5 to 86.2) and 79.0 (95% CI: 78.9 to 79.2), respectively. From 1999 to 2001, AAMRs increased sharply in both urban (APC: 57.93; 95% CI: −4.65 to 161.58) and rural (APC: 54.22; 95% CI: 29.41 to 83.78) areas. After 2001, urban areas experienced a steady rise in AAMR through 2020 (APC: 3.16; 95% CI: 2.40 to 3.92), while rural areas followed a more variable trend: a significant increase from 2001 to 2008 (APC: 6.59; 95% CI: 4.84 to 8.38), a slower rise from 2008 to 2018 (APC: 1.74; 95% CI: 0.99 to 2.49), and a sharp increase again from 2018 to 2020 (APC: 16.26; 95% CI: 8.52 to 24.54). The rural-urban mortality gap widened modestly from 6.5 per 100,000 in 1999 to 7.8 per 100,000 in 2020, reflecting persistent but stable geographic inequity (Fig. 6; Tables S3 and S9, Supplemental Digital Content, https://links.lww.com/MD/Q920).

### 3.6. Sensitivity analyses

Stratified analyses showed consistent findings across most subgroups. Both genders exhibited significant rising mortality, with a steeper increase among males (AAPC: 4.59 [95% CI: 2.63 to 6.60]) compared to females (AAPC: 3.03 [95% CI: 0.68 to 5.44]). Across racial and ethnic groups, significant increases were observed among Hispanic or Latino (AAPC: 2.65 [95% CI: 1.62 to 3.69]) and Asian or Pacific Islander (AAPC: 4.48 [95% CI: 1.27 to 7.79]) populations. The upward trends for Black or African American (AAPC: 1.95 [95% CI: −0.69 to 4.66]), White (AAPC: 2.86 [95% CI: −0.12 to 5.93]), and American Indian or Alaska Native (AAPC: 2.93 [95% CI: −0.51 to 6.49]) populations were not statistically significant.

Age-specific analysis revealed significant increases across all older adult groups. The most pronounced rise was observed among those aged 85 years and older (AAPC: 3.83 [95% CI: 1.25 to 6.47]), followed by the 65- to 74-year age group (AAPC: 3.63 [95% CI: 1.56 to 5.74]). A significant increase was also sustained in the 75- to 84-year age group (AAPC: 2.48 [95% CI: 0.03 to 4.99]).

Regionally, mortality increased significantly in the South (AAPC: 3.05 [95% CI: 0.58 to 5.58]) and the West (AAPC: 3.32 [95% CI: 1.39 to 5.28]). The Northeast (AAPC: 3.33 [95% CI: −0.25 to 7.04]) and Midwest (AAPC: 2.70 [95% CI: −0.23 to 5.73]) exhibited upward trends that did not reach statistical significance. Both urban and rural areas showed significant upward trends, with rural regions exhibiting a nominally greater rise (AAPC: 3.98 [95% CI: 1.73 to 6.28]) compared to urban regions (AAPC: 3.48 [95% CI: 1.08 to 5.93]).

These findings confirm that the significant increasing mortality trends persisted when using a more specific case definition, demonstrating the robustness of the main results (Table S10, Supplemental Digital Content, https://links.lww.com/MD/Q920).

## 4. Discussion

We identified several key findings in our analysis of HTN and dementia-related mortality trends in the US from 1999 to 2020 using CDC WONDER data. Overall, the AAMR rose significantly over the study period, with the steepest increases occurring between 1999 and 2001 and 2018 and 2020. Both males and females exhibited rising trends, although mortality was consistently higher among females. When stratified by race, NH Black or African American individuals had the highest AAMR. Geographically, states like Mississippi, Vermont, Ohio, and Texas ranked in the top 90th percentile for mortality, while Massachusetts, Nevada, Florida, and Arizona were in the lowest 10th percentile. Mortality was consistently elevated in nonmetropolitan regions, indicating urban-rural disparities. These patterns underscore a growing public health burden posed by coexisting HTN and dementia, particularly among vulnerable populations.

HTN and dementia are frequently co-reported on death certificates and have biologically plausible links, but our population-level, observational design cannot establish causality. Prior literature associates long-standing hypertension with cerebrovascular injury and neurodegenerative changes, including small-vessel disease, hypoperfusion, and blood–brain barrier dysfunction, which may contribute to cognitive decline. Experimental and longitudinal data also suggest relationships with amyloid and tau pathology; however, these pathways should be interpreted as potential mechanisms rather than causal proof in the context of our study design.^[14]^

Our observed mortality trends align with previous research on vascular risk factors and cognitive decline.^[15,16]^ The sharp rise in mortality from 1999 to 2001 may be partially attributed to improved diagnostic recognition of dementia and greater reporting accuracy on death certificates.^[17]^ The more gradual increase from 2001 to 2011 corresponds with demographic shifts, particularly the aging population with higher prevalence of HTN and dementia.^[18]^ Between 2011 and 2018, mortality plateaued, but a steep rise followed from 2018 to 2020. This recent increase likely reflects a confluence of factors: population aging, growing racial and socioeconomic disparities in healthcare access,^[19,20]^ a rising prevalence of midlife HTN,^[21]^ and increasing rates of multimorbidity, including obesity, diabetes, and metabolic syndrome that complicate HTN management and elevate dementia risk.^[22–24]^ Emerging evidence also links environmental toxins and air pollution to neurodegenerative diseases.^[25]^ Additionally, a study by Wang et al in 2021 suggests that COVID-19 exacerbated dementia mortality in 2020.^[26]^

The observed sex-based differences with higher AAMRs in women are consistent with prior findings showing greater dementia prevalence among females, particularly in older age groups. Beam et al in 2018 reported that dementia, especially Alzheimer disease, was more common in women over age 80.^[27]^ Higher female mortality at older ages may reflect greater longevity and hypothesized sex-specific biological susceptibility; these factors were not measurable in our data.^[28,29]^ Although men had lower absolute mortality rates, their AAPC was comparable, suggesting that both sexes are affected by the rising trend.

Our findings also underscore racial disparities, with NH Black individuals experiencing the highest mortality rates, followed by NH Whites and Hispanics. While NH Asians or Pacific Islanders had the lowest AAMR, all racial groups experienced an increase over time. These disparities align with prior studies documenting greater cardiovascular and dementia burden among racial minorities.^[30–32]^ African Americans often develop HTN earlier, with more severe progression and poorer control, heightening the risk of vascular dementia.^[33]^ Mayeda et al in 2016 found a persistently elevated dementia incidence in Black adults, even after adjusting for socioeconomic factors.^[32]^ These disparities reflect systemic inequities in healthcare access, chronic disease management, and social determinants of health.^[34]^ Notably, the absolute gap between the highest‐ and lowest-burden groups widened over time, reinforcing the need to examine upstream social and structural drivers.

The most pronounced increases in mortality were seen among adults aged 85 and older, highlighting the interplay between aging, vascular risk, and cognitive decline. Midlife HTN has been shown to elevate dementia risk in later life due to cumulative cerebrovascular damage.^[16]^ For instance, a JAMA study reported a 49% higher dementia risk in individuals with sustained HTN across midlife and late life.^[35]^ Similarly, the Whitehall II study found that HTN beginning at age 50 was associated with a 38% higher dementia risk, emphasizing the importance of early BP control.^[36]^

Geographic variation in AAMR was also notable, with states in the South and Midwest, especially those within the Stroke Belt, reporting the highest rates. State-level variation mirrored known regional cardiovascular risk patterns.^[37]^ Individuals born in the Stroke Belt have a higher lifetime risk of cognitive impairment and dementia-related mortality, particularly among African Americans.^[38]^ Higher mortality rates in rural areas compared to urban areas further reinforce the impact of geographic inequities. Cross et al in 2021 reported that by 2018, rural regions had the highest Alzheimer disease and related dementia AAMR.^[39]^ These rural-urban differences are shaped by limited access to healthcare, lower educational attainment, social isolation, and higher prevalence of chronic diseases in nonmetropolitan communities.^[40]^ These regional patterns should be interpreted as contextual associations that align with known geographic cardiovascular risk gradients, not as causal effects of residence alone.

These findings have critical public health and policy implications. The increasing burden of hypertension and dementia-related mortality warrants enhanced preventive strategies, including improved hypertension screening, aggressive BP control, and lifestyle modifications.^[41]^ Additionally, addressing healthcare disparities through policy changes, including improved access to primary care, community-based interventions, and culturally tailored health education, may help reduce racial and regional mortality disparities.^[22]^

Our findings highlight populations and regions with the highest mortality burden, supporting equity-focused prevention: aggressive midlife blood-pressure control, improved access to primary care in rural and high-burden states, and culturally tailored community programs. Future research should link mortality to individual-level clinical and social data to quantify the roles of comorbidities, healthcare access, and social determinants, and reassess post-2020 trends using a consistent denominator framework (e.g., harmonized single-race series or bridged solutions).

### 4.1. Strengths

This nationwide analysis uses finalized CDC WONDER multiple-cause-of-death data (1999 to 2020), enabling stable long-term trend estimation across demographic and geographic strata. The extended study period enables a comprehensive examination of trends in vascular dementia, Alzheimer, and HTN using the robust CDC WONDER database. This, in turn, facilitates in-depth stratification by key demographic factors such as age, sex, and race, providing critical insights into disparities in disease prevalence and mortality and pinpointing areas where targeted public health interventions may be most needed.

### 4.2. Limitations

However, several limitations must be acknowledged. The potential underestimation of mortality rates in the CDC WONDER database, stemming from its reliance on the precise reporting and coding of death certificates, represents a notable constraint. Some cases go unreported, and the cause of death from out-of-hospital reported deaths is less reliable, so causal conclusions cannot be drawn from these results. Moreover, because the CDC mortality data used in this study were aggregated, there is a risk that some fatalities were inaccurately recorded, misclassified, or otherwise reported improperly. Due to the suppression criteria of CDC Wonder, deaths under a reliable margin were not accessible for the year 1999 for the American Indian or Alaska Native population group. The study did not examine the underlying causes or influencing factors contributing to the observed racial or sex-based disparities in mortality rates. Furthermore, shifts in healthcare practices and societal factors that occurred before or after the study period may not be fully accounted for, which can influence the reliability of the trend analysis. Analyses were restricted to 1999 to 2020 to preserve continuity in bridged-race denominators. The finalized 2018 to 2023 series uses single-race denominators and documents county-level constraints for Urbanization categories, complicating cross-series urban–rural and race/ethnicity trend comparisons.

## 5. Conclusions

In this nationwide observational analysis of finalized CDC WONDER data (1999–2020), hypertension- and dementia-related age-adjusted mortality increased over time, with higher burdens among adults 85 years and older, women, NH Black individuals, and residents of rural and Southern regions. These descriptive trends indicate persistent demographic and geographic disparities. Findings may inform equity-focused public health planning, particularly improved blood-pressure control and access to care in high-burden communities. Future studies linking mortality with individual-level clinical and social data, and analyses incorporating finalized post-2020 files, are needed to confirm and further characterize these patterns.

## Acknowledgments

All authors would like to extend their sincere regards to the team of the Research Council of Pakistan (RCOP) for their guidance and mentorship.

## Author contributions

**Conceptualization:** Asad Ali Ahmed Cheema.

**Data curation:** Muhammad Ibrahim, Umair Iqbal.

**Formal analysis:** Muhammad Ibrahim.

**Investigation:** Muhammad Ibrahim.

**Project administration:** Asad Ali Ahmed Cheema, Areen Zia.

**Supervision:** Asad Ali Ahmed Cheema.

**Visualization:** Muhammad Ibrahim, Areen Zia, Umair Iqbal, Rameesha Babar.

**Writing – original draft:** Asad Ali Ahmed Cheema, Areen Zia, Ahmed Hasan, Rameesha Babar, Muhammad Moghis, Hurriyah Akbar, Tayyaba Shahiq, Asma Rizwan.

**Writing – review & editing:** Asad Ali Ahmed Cheema, Muhammad Ibrahim, Areen Zia, Umair Iqbal, Ahmed Hasan, Muhammad Moghis, Hurriyah Akbar.

## Supplementary Material


