# Supplementary material for: Trends in hypertension- and dementia-related mortality in the United States: An observational analysis from 1999 to 2020
Source: Medicine (Baltimore). 2025 Dec 19;104(51):e46611. doi: 10.1097/MD.0000000000046611 (PMC12727369; doi:10.1097/MD.0000000000046611)
Supplement: Supplementary file 1 [file medi-104-e46611-s001.pdf]

Supplemental Figure 1: Cohort assembly (1999–2020): decedents ≥55 years with both Hypertension (I10–I15) and dementia (F01/F03/G30) as underlying or contributing causes; excluded single-condition records, age <55, and missing/suppressed demographics. AAMRs (2000 U.S. standard) estimated; trends via Joinpoint (APC/AAPC); sensitivity required hypertension as underlying cause.

Abbreviations: AAMR, age-adjusted mortality rate; APC, annual percent change; AAPC, average annual percent change; ICD-10, International Classification of Diseases, 10th Revision; NCHS, National Center for Health Statistics.

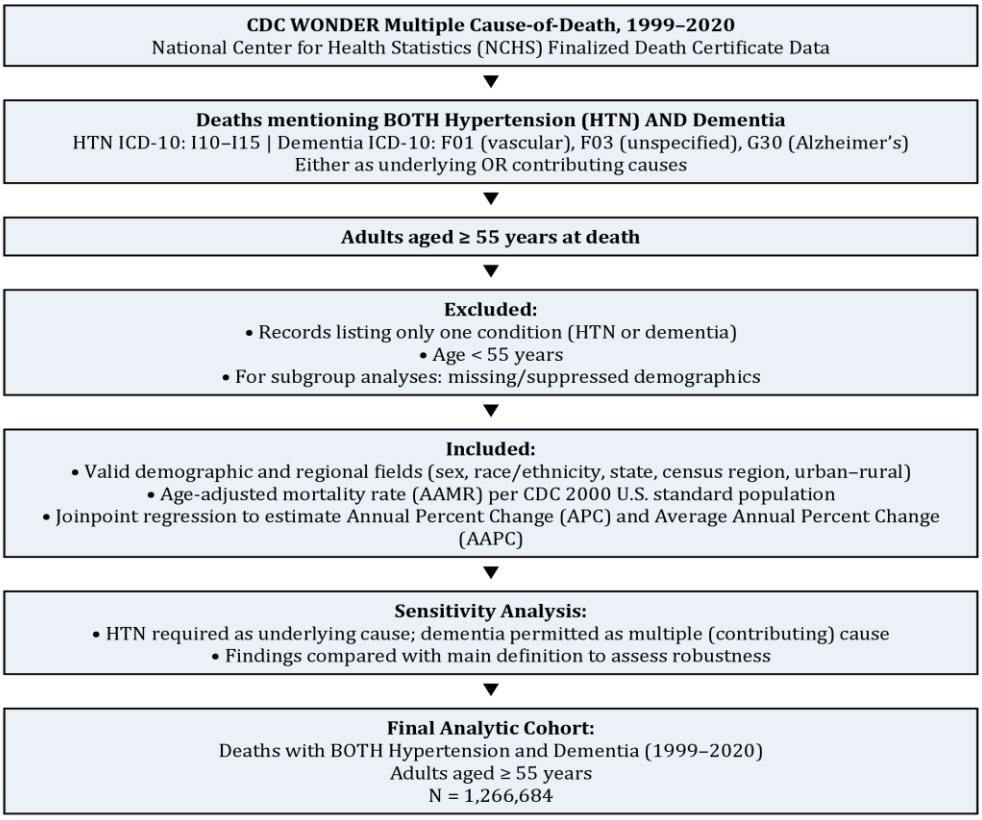

Supplemental Figure 2: Trends in Hypertension- and Dementia-Related Mortality in the United States, 1999–2020. Age-adjusted mortality rates (AAMRs) per 100,000 population are shown for all decedents with both hypertension and dementia listed as causes of death. AAMR = age-adjusted mortality rate; AAPC = average annual percent change; CI = confidence interval.

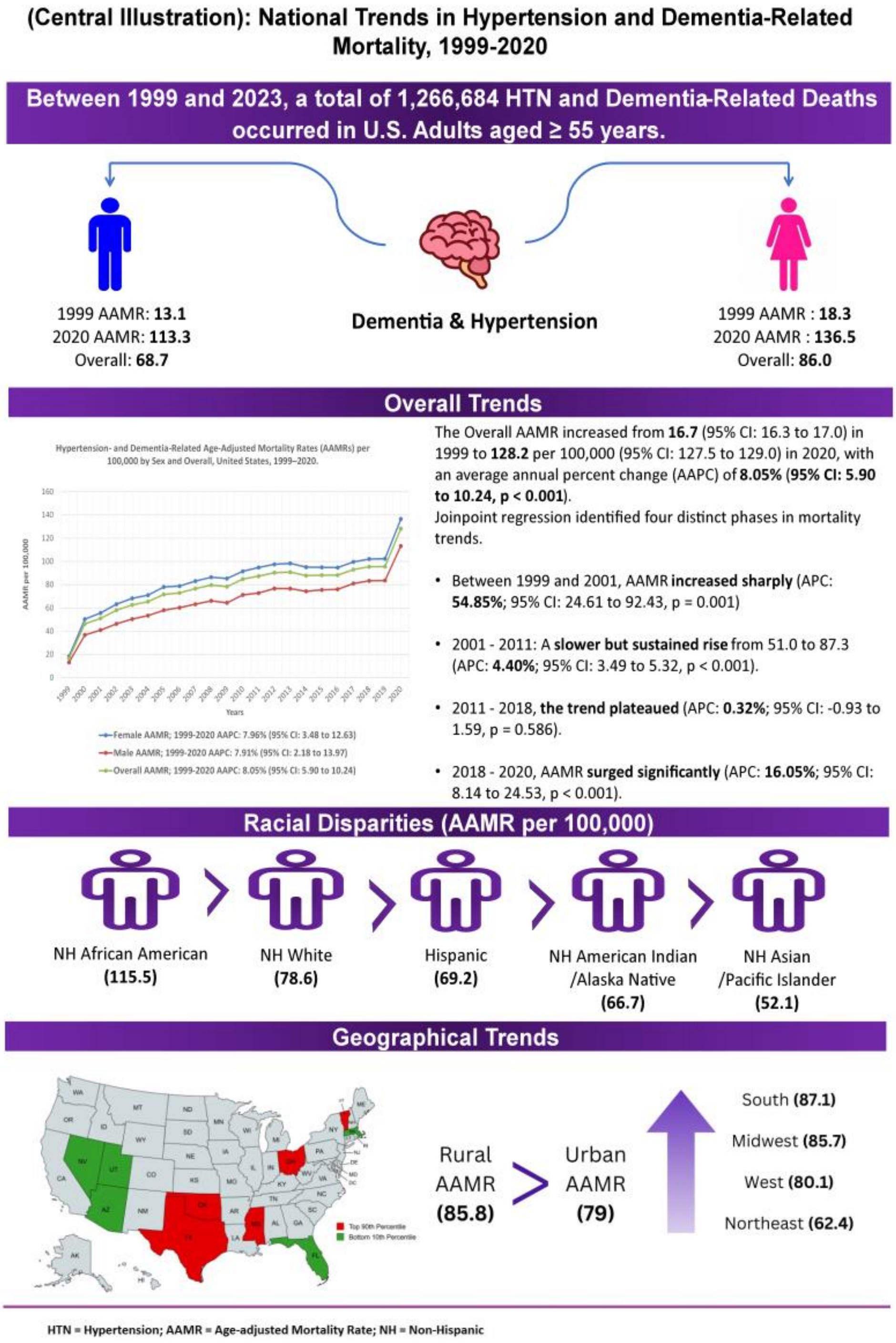

Supplemental Table 1: Hypertension and Dementia related Deaths, Stratified by Sex and Race, in Adults in the United States, 1999 to 2020.

Year	Overall	Women	Men	NH White	NH Black or African American	NH Asian or Pacific Islander	NH American Indian or Alaska Native	Hispanic or Latino	Population
**1999**	9,614	7,028	2,586	8,056	1,199	79	13	240	58,575,867
**2000**	27,121	19,660	7,461	22,701	3,277	346	45	689	59,266,437
**2001**	30,426	21,986	8,440	25,372	3,699	378	55	842	60,395,586
**2002**	35,012	25,245	9,767	29,172	4,228	442	83	986	62,225,539
**2003**	38,497	27,617	10,880	32,142	4,548	487	107	1,138	63,872,474
**2004**	40,787	29,021	11,766	33,642	4,960	619	100	1,387	65,508,623
**2005**	45,683	32,538	13,145	37,776	5,413	687	126	1,615	67,291,295
**2006**	47,648	33,597	14,051	39,445	5,423	803	132	1,787	69,094,220
**2007**	51,238	36,073	15,165	42,471	5,841	866	151	1,856	70,954,145

**2008**	54,485	38,171	16,314	45,216	6,044	904	123	2,103	72,934,684
**2009**	54,676	38,342	16,334	44,849	6,124	1,092	180	2,315	75,028,775
**2010**	60,349	41,912	18,437	49,573	6,589	1,144	189	2,758	76,750,713
**2011**	64,226	44,581	19,645	52,655	6,940	1,241	181	3,106	79,456,281
**2012**	67,995	46,675	21,320	55,365	7,351	1,447	227	3,466	81,731,558
**2013**	70,013	47,969	22,044	56,681	7,528	1,494	216	3,953	84,020,505
**2014**	69,211	47,187	22,024	55,889	7,406	1,463	269	4,028	86,320,792
**2015**	71,016	48,033	22,983	57,250	7,488	1,608	265	4,204	88,638,671
**2016**	72,373	48,597	23,776	58,130	7,725	1,689	258	4,421	90,707,339
**2017**	77,897	51,813	26,084	62,429	8,312	1,927	312	4,747	92,854,337
**2018**	81,674	54,008	27,666	65,338	8,418	2,195	361	5,203	94,703,829
**2019**	83,326	54,744	28,582	66,444	8,740	2,225	307	5,475	96,506,800
**2020**	113,417	73,756	39,661	87,521	13,225	3,254	493	8,704	98,063,042
**Total**	1,266,684	868,553	398,131	1,028,117	140,478	26,390	4,193	65,023	1,694,901,512

*NH, non-Hispanic.

Supplemental Table 2: Hypertension and Dementia related Mortality Stratified by Place of Death in Adults in the United States, 1999 to 2020.

Year	Medical Facility	Nursing Home/Long-term Care Facility	Hospices	Home
**1999**	2,175	6,016	Missing	1,150
**2000**	6,257	16,920	Missing	3,113
**2001**	6,736	19,021	Missing	3,601
**2002**	7,767	21,665	Missing	4,227
**2003**	8,098	23,607	38	5,021
**2004**	8,253	24,681	98	5,747
**2005**	9,249	27,377	411	6,482
**2006**	9,232	28,334	601	7,151
**2007**	9,733	30,052	1,047	7,827
**2008**	10,092	31,287	1,308	8,524
**2009**	9,604	30,335	1,349	9,330
**2010**	10,339	33,727	1,859	10,929
**2011**	10,614	35,480	2,351	11,893
**2012**	10,559	37,112	2,701	13,067
**2013**	10,432	37,965	3,021	13,840
**2014**	9,961	38,085	3,070	13,826
**2015**	10,107	38,893	3,438	14,335
**2016**	9,953	38,517	3,727	15,308
**2017**	10,375	41,530	4,233	16,402
**2018**	10,463	43,255	4,560	17,904
**2019**	10,623	42,749	5,257	18,920

**2020**	16,499	54,789	6,688	27,426
**Total**	207,121	701,397	45,757	236,023

Supplemental Table 3 Annual percent change (APC) of Hypertension and Dementia Age-Adjusted Mortality Rates per 100,000 in Adults in the United States, 1999 to 2020.

Year Interval	APC (95% CI)
**Overall**	
1999-2001	54.85 (24.60 to 92.43)
2001-2011	4.39 (3.48 to 5.31)
2011-2018	0.32 (-0.93 to 1.58)
2018-2020	16.04 (8.13 to 24.53)
**Men**	
1999-2001	59.25 (-13.71 to 193.94)
2001-2020	3.57 (2.80 to 4.36)
**Women**	
1999-2001	70.14 (5.86 to 173.45)
2005-2020	2.90 (2.19 to 3.61)
**NH White**	
1999-2001	58.52 (-2.28 to 157.15)
2001-2020	3.51 (2.85 to 4.18)
**NH Black or African American**	
1999-2001	61.59 (32.99 to 96.35)
2001-2011	2.94 (1.89 to 4.00)
2011-2018	-1.78 (-3.29 to -0.25)
2018-2020	21.47 (12.21 to 31.48)
**NH American Indian or Alaska Native**	
2000-2003	33.26 (-6.43 to 89.81)

2003-2020	2.96 (1.60 to 4.34)
**Hispanic or Latino**	
1999-2001	60.90 (18.84 to 117.84)
2001-2012	6.39 (5.24 to 7.55)
2012-2018	-0.96 (-2.96 to 1.06)
2018-2020	25.74 (16.86 to 35.29)
**NH Asian or Pacific Islander**	
1999-2006	11.86 (3.92 to 20.41)
2006-2018	0.09 (-1.78 to 2.01)
2018-2020	16.65 (-4.49 to 42.49)
**Metropolitan areas**	
1999-2001	57.92 (-4.65 to 161.57)
2001-2020	3.15 (2.40 to 3.91)
**Non-metropolitan areas**	
1999-2001	54.21 (29.40 to 83.78)
2001-2008	6.59 (4.83 to 8.37)
2008-2018	1.74 (0.99 to 2.49)
2018-2020	16.25 (8.52 to 24.54)

*APC = annual percent change; NH = non-Hispanic.

Supplemental Table 4: Overall and Sex Stratified Hypertension and Dementia related Age-Adjusted Mortality Rates per 100,000 in Adults in the United States, 1999 to 2020.

Year	Men	Women	Overall
**1999**	13.1 (12.5-13.6)	18.3 (17.8-18.7)	16.7 (16.3-17.0)
**2000**	36.7 (35.9-37.6)	50.3 (49.6-51.0)	46.2 (45.7-46.8)
**2001**	40.9 (40.0-41.8)	55.6 (54.9-56.3)	51.0 (50.4-51.6)
**2002**	46.3 (45.4-47.2)	63.2 (62.4-64.0)	58.0 (57.4-58.6)
**2003**	50.4 (49.4-51.3)	68.2 (67.4-69.0)	62.6 (62.0-63.3)
**2004**	53.4 (52.4-54.4)	71.0 (70.2-71.8)	65.4 (64.8-66.1)
**2005**	58.0 (57.0-59.0)	78.0 (77.1-78.8)	71.6 (71.0-72.3)
**2006**	60.2 (59.2-61.2)	78.8 (78.0-79.7)	72.9 (72.3-73.6)
**2007**	63.2 (62.2-64.2)	83.1 (82.2-84.0)	76.6 (75.9-77.2)
**2008**	66.1 (65.1-67.2)	86.4 (85.5-87.3)	79.7 (79.0-80.3)
**2009**	64.4 (63.4-65.4)	85.3 (84.4-86.1)	78.3 (77.6-

			78.9)
**2010**	71.2 (70.2-72.3)	91.5 (90.6-92.4)	84.8 (84.1-85.4)
**2011**	72.8 (71.8-73.8)	94.8 (93.9-95.7)	87.3 (86.6-88.0)
**2012**	76.7 (75.6-77.7)	97.5 (96.6-98.4)	90.3 (89.6-90.9)
**2013**	76.6 (75.6-77.6)	98.3 (97.4-99.2)	90.8 (90.1-91.4)
**2014**	74.3 (73.3-75.3)	95.1 (94.2-96.0)	87.8 (87.2-88.5)
**2015**	75.5 (74.5-76.5)	94.9 (94.0-95.8)	88.2 (87.5-88.8)
**2016**	76.0 (75.0-77.0)	94.7 (93.8-95.6)	88.2 (87.5-88.8)
**2017**	81.0 (80.0-82.0)	99.6 (98.8-100.5)	93.0 (92.3-93.6)
**2018**	83.3 (82.3-84.3)	102.1 (101.3-103.0)	95.4 (94.8-96.1)
**2019**	83.6 (82.6-84.5)	102.3 (101.5-103.2)	95.6 (94.9-96.2)
**2020**	113.3 (112.1-114.4)	136.5 (135.5-137.5)	128.2 (127.5-129.0)

Supplemental Table 5: Hypertension and Dementia related Crude Mortality Rates per 100,000, Stratified by Age-Groups in Adults in the United States, 1999 to 2020.

Year	Age 55-64	Age 65-74	Age 75-44	Age 85+
1999	0.2	3.1	27.1	136.7
2000	0.9	9.2	73.4	380.6
2001	0.9	9.9	80.1	424
2002	0.9	11.1	90.5	484.7
2003	1.1	12	97.3	524.1
2004	1.1	12.2	102.1	548.1
2005	1.3	12.9	110.5	605
2006	1.3	13.1	111.6	618.8
2007	1.4	13.5	117.9	648.3
2008	1.3	14.1	122.6	675.6
2009	1.2	13.9	121.3	661.7
2010	1.4	14.1	128.9	726.9
2011	1.5	15.1	133.6	743.5
2012	1.6	15.1	137.1	773.6
2013	1.7	15.3	137.5	778.1
2014	1.6	14.9	132	755.7
2015	1.5	14.8	130.2	766.7
2016	1.5	15.6	129.5	765.6
2017	1.6	16.5	135.6	809.5
2018	1.8	17	139.1	830.1
2019	1.8	17.5	139.7	828.3
2020	2.5	24.4	189.9	1099.3

Supplemental Table 6: Hypertension and Dementia related Age-Adjusted Mortality Rates per 100,000, Stratified by Race in Adults in the United States, 1999 to 2020.

Year	NH White	NH Black or African American	NH American Indian or Alaska Native	Hispanic or Latino	NH Asian or Pacific Islander
**1999**	16.1 (15.8-16.5)	27.5 (25.9-29.1)	-	11.1 (9.7-12.5)	8.0 (6.3-10.1)
**2000**	44.7 (44.2-45.3)	74.2 (71.6-76.7)	24.0 (17.5-32.1)	29.8 (27.5-32.0)	32.7 (29.2-36.2)
**2001**	49.3 (48.7-49.9)	83.1 (80.4-85.8)	28.0 (21.1-36.6)	34.6 (32.2-36.9)	32.2 (28.9-35.5)
**2002**	56.1 (55.4-56.7)	94.8 (91.9-97.7)	41.6 (33.0-51.7)	38.5 (36.0-40.9)	35.6 (32.3-39.0)
**2003**	60.8 (60.2-61.5)	101.0 (98.1-104.0)	52.5 (42.4-62.7)	42.6 (40.1-45.2)	36.5 (33.2-39.8)
**2004**	63.0 (62.3-63.7)	108.8 (105.8-111.9)	47.1 (37.6-56.5)	48.7 (46.1-51.3)	44.1 (40.6-47.6)
**2005**	69.4 (68.7-70.1)	115.7 (112.6-118.8)	58.9 (48.5-69.3)	53.6 (51.0-56.2)	45.2 (41.8-48.6)
**2006**	70.9 (70.2-71.6)	113.6 (110.5-116.6)	58.8 (48.6-69.0)	56.2 (53.5-58.8)	49.0 (45.6-52.4)
**2007**	74.8 (74.1-75.5)	119.1 (116.1-122.2)	65.4 (54.8-76.1)	55.2 (52.7-57.7)	49.9 (46.6-53.3)
**2008**	78.2 (77.5-79.0)	120.2 (117.1-123.2)	51.7 (42.4-61.0)	58.9 (56.4-61.5)	49.0 (45.8-52.2)

**2009**	76.3	118.4	74.4	60.9	55.5
	(75.6-77.0)	(115.4-121.4)	(63.4-85.4)	(58.4-63.4)	(52.2-58.8)
**2010**	83.0	123.8	73.8	69.8	55.6
	(82.3-83.8)	(120.8-126.9)	(63.1-84.5)	(67.2-72.4)	(52.3-58.8)
**2011**	85.9	126.1	66.4	71.4	54.1
	(85.2-86.7)	(123.1-129.0)	(56.6-76.2)	(68.9-73.9)	(51.1-57.2)
**2012**	88.9	127.8	77.8	74.7	58.4
	(88.1-89.6)	(124.9-130.8)	(67.5-88.0)	(72.2-77.2)	(55.3-61.4)
**2013**	89.5	126.6	68.5	79.6	55.2
	(88.7-90.2)	(123.7-129.4)	(59.2-77.8)	(77.1-82.1)	(52.4-58.0)
**2014**	87.0	120.3	80.7	75.7	50.2
	(86.3-87.7)	(117.5-123.0)	(70.9-90.5)	(73.3-78.0)	(47.7-52.8)
**2015**	87.9	117.3	75.6	73.8	50.8
	(87.2-88.6)	(114.6-119.9)	(66.4-84.8)	(71.6-76.1)	(48.3-53.3)
**2016**	88.2	117.4	66.4	73.7	50.3
	(87.5-88.9)	(114.8-120.1)	(58.2-74.7)	(71.5-75.9)	(47.9-52.7)
**2017**	93.6	121.7	78.4	74.4	53.2
	(92.9-94.3)	(119.1-124.4)	(69.6-87.2)	(72.2-76.5)	(50.8-55.5)
**2018**	96.5	119.3	85.3	77.8	57.3
	(95.7-97.2)	(116.7-121.9)	(76.4-94.2)	(75.7-80.0)	(54.9-59.7)
**2019**	96.9	119.7	68.8	78.8	54.7
	(96.2-97.7)	(117.2-122.2)	(61.0-76.6)	(76.7-80.9)	(52.4-57.0)
**2020**	126.7	175.7	103.0	120.1	75.3
	(125.9-127.6)	(172.7-178.8)	(93.8-112.2)	(117.5-122.6)	(72.7-77.9)

***NH = non-Hispanic.

Supplemental Table 7: Hypertension and Dementia related Age-Adjusted Mortality Rates per 100,000, Stratified by States in Adults in the United States, 1999 to 2020.

State	Age-Adjusted Rate (95% CI)
Alabama	73.9 (72.8-75)
Alaska	68.4 (64.3-72.6)
Arizona	54 (53.2-54.8)
Arkansas	80 (78.6-81.5)
California	86.9 (86.5-87.4)
Colorado	83.9 (82.6-85.2)
Connecticut	57.9 (56.9-58.9)
Delaware	86.9 (84.2-89.5)
District of Columbia	96.7 (93.1-100.3)
Florida	52.8 (52.4-53.2)
Georgia	77.8 (76.9-78.7)
Hawaii	66.6 (64.8-68.4)
Idaho	68.1 (66.2-70)
Illinois	65.6 (65-66.3)
Indiana	96.4 (95.4-97.5)
Iowa	72.8 (71.6-74)

Kansas	57.2 (56-58.3)
Kentucky	88.1 (86.9-89.4)
Louisiana	78.3 (77.1-79.5)
Maine	58.4 (56.8-60.1)
Maryland	107.1 (105.9-108.3)
Massachusetts	48.6 (47.9-49.3)
Michigan	81.2 (80.5-82)
Minnesota	114.9 (113.6-116.1)
Mississippi	135 (133.1-137)
Missouri	68.4 (67.5-69.3)
Montana	67.8 (65.6-69.9)
Nebraska	98.4 (96.5-100.3)
Nevada	51.3 (49.9-52.8)
New Hampshire	72.8 (70.8-74.8)
New Jersey	65.4 (64.7-66.2)
New Mexico	67.1 (65.5-68.8)
New York	61.3 (60.9-61.8)
North Carolina	95.6 (94.7-96.5)
North Dakota	97.6 (94.7-100.5)

Ohio	114.9 (114.1-115.8)
Oklahoma	137 (135.3-138.6)
Oregon	100 (98.7-101.4)
Pennsylvania	64.2 (63.7-64.8)
Rhode Island	90.7 (88.4-92.9)
South Carolina	108.7 (107.3-110.1)
South Dakota	76.5 (74.1-78.9)
Tennessee	106.5 (105.4-107.7)
Texas	110.4 (109.7-111.1)
Utah	46.9 (45.5-48.3)
Vermont	123.7 (120.1-127.4)
Virginia	69.7 (68.9-70.6)
Washington	87.2 (86.2-88.2)
West Virginia	102.5 (100.6-104.4)
Wisconsin	73.2 (72.3-74.1)
Wyoming	62.7 (59.6-65.7)

Supplemental Table 8: Vascular Dementia and Hypertension Age-Adjusted Mortality Rates per 100,000, Stratified by Census Region in Adults in the United States, 1999 to 2020.

Census Region	Year	Age-Adjusted Rate (95% CI)
**Northeast**	1999	12.9 (12.3-13.6)
**Northeast**	2000	36.8 (35.8-37.9)
**Northeast**	2001	38.4 (37.4-39.5)
**Northeast**	2002	45.3 (44.1-46.4)
**Northeast**	2003	45.5 (44.4-46.7)
**Northeast**	2004	49.8 (48.6-50.9)
**Northeast**	2005	53.6 (52.4-54.9)
**Northeast**	2006	53.9 (52.7-55.2)
**Northeast**	2007	57.3 (56.0-58.5)
**Northeast**	2008	58.5 (57.2-59.7)
**Northeast**	2009	58.0 (56.7-59.2)
**Northeast**	2010	66.6 (65.3-67.9)
**Northeast**	2011	70.3 (69.0-71.7)
**Northeast**	2012	71.4 (70.1-72.8)
**Northeast**	2013	71.6 (70.3-73.0)
**Northeast**	2014	70.9 (69.5-72.2)
**Northeast**	2015	71.4 (70.1-72.7)
**Northeast**	2016	70.6 (69.3-71.9)
**Northeast**	2017	74.4 (73.1-75.8)
**Northeast**	2018	78.7 (77.4-80.1)
**Northeast**	2019	75.0 (73.7-76.3)

**Northeast**	2020	106.2 (104.7-107.8)
**Northeast**	**Total**	62.4 (62.1-62.7)
**Midwest**	1999	17.5 (16.8-18.2)
**Midwest**	2000	50.8 (49.6-52.0)
**Midwest**	2001	56.1 (54.9-57.3)
**Midwest**	2002	62.1 (60.8-63.4)
**Midwest**	2003	68.6 (67.3-69.9)
**Midwest**	2004	70.6 (69.3-72.0)
**Midwest**	2005	78.4 (77.0-79.8)
**Midwest**	2006	79.2 (77.8-80.6)
**Midwest**	2007	84.5 (83.1-85.9)
**Midwest**	2008	87.7 (86.2-89.1)
**Midwest**	2009	85.5 (84.1-87.0)
**Midwest**	2010	88.8 (87.3-90.2)
**Midwest**	2011	93.0 (91.5-94.4)
**Midwest**	2012	96.3 (94.8-97.7)
**Midwest**	2013	95.9 (94.4-97.3)
**Midwest**	2014	95.0 (93.5-96.4)
**Midwest**	2015	95.6 (94.2-97.0)
**Midwest**	2016	96.2 (94.7-97.6)
**Midwest**	2017	101.1 (99.7-102.6)
**Midwest**	2018	101.3 (99.9-102.8)
**Midwest**	2019	101.5 (100.0-102.9)
**Midwest**	2020	137.2 (135.6-138.9)
**Midwest**	**Total**	85.7 (85.4-86.0)

**South**	1999	18.4 (17.8-19.0)
**South**	2000	47.6 (46.6-48.5)
**South**	2001	53.3 (52.3-54.3)
**South**	2002	61.7 (60.6-62.8)
**South**	2003	67.3 (66.2-68.4)
**South**	2004	69.5 (68.4-70.7)
**South**	2005	76.8 (75.6-78.0)
**South**	2006	77.1 (75.9-78.2)
**South**	2007	82.5 (81.3-83.7)
**South**	2008	85.2 (84.1-86.4)
**South**	2009	85.2 (84.1-86.4)
**South**	2010	93.3 (92.1-94.5)
**South**	2011	93.8 (92.6-95.0)
**South**	2012	97.1 (95.9-98.3)
**South**	2013	98.8 (97.7-100.0)
**South**	2014	94.5 (93.4-95.7)
**South**	2015	94.1 (93.0-95.2)
**South**	2016	93.3 (92.2-94.4)
**South**	2017	99.7 (98.5-100.8)
**South**	2018	103.2 (102.1-104.3)
**South**	2019	105.6 (104.5-106.7)
**South**	2020	142.6 (141.3-143.9)
**South**	**Total**	87.1 (86.8-87.3)
**West**	1999	16.8 (16.0-17.5)
**West**	2000	48.7 (47.4-50.0)

**West**	2001	54.5 (53.1-55.8)
**West**	2002	60.4 (59.0-61.8)
**West**	2003	65.9 (64.5-67.4)
**West**	2004	68.9 (67.5-70.4)
**West**	2005	73.7 (72.2-75.2)
**West**	2006	78.1 (76.5-79.6)
**West**	2007	77.3 (75.8-78.8)
**West**	2008	82.7 (81.2-84.2)
**West**	2009	78.5 (77.1-80.0)
**West**	2010	83.9 (82.4-85.3)
**West**	2011	86.6 (85.1-88.1)
**West**	2012	90.0 (88.5-91.5)
**West**	2013	89.8 (88.3-91.3)
**West**	2014	84.6 (83.2-86.0)
**West**	2015	85.5 (84.1-86.9)
**West**	2016	86.9 (85.6-88.3)
**West**	2017	90.1 (88.7-91.4)
**West**	2018	91.0 (89.7-92.4)
**West**	2019	90.9 (89.5-92.3)
**West**	2020	114.1 (112.6-115.6)
**West**	**Total**	80.1 (79.8-80.4)
**Total**	**Total**	80.2 (80.1-80.4)
